# Specific Features of RNA Polymerases I and III: Structure and Assembly

**DOI:** 10.3389/fmolb.2021.680090

**Published:** 2021-05-14

**Authors:** Tomasz W. Turowski, Magdalena Boguta

**Affiliations:** ^1^Wellcome Trust Centre for Cell Biology, University of Edinburgh, Edinburgh, United Kingdom; ^2^Institute of Biochemistry and Biophysics, Polish Academy of Sciences, Warsaw, Poland

**Keywords:** RNA polymerase I, RNA polymerase III, complex assembly, transcription factors, tRNA, rRNA

## Abstract

RNA polymerase I (RNAPI) and RNAPIII are multi-heterogenic protein complexes that specialize in the transcription of highly abundant non-coding RNAs, such as ribosomal RNA (rRNA) and transfer RNA (tRNA). In terms of subunit number and structure, RNAPI and RNAPIII are more complex than RNAPII that synthesizes thousands of different mRNAs. Specific subunits of the yeast RNAPI and RNAPIII form associated subcomplexes that are related to parts of the RNAPII initiation factors. Prior to their delivery to the nucleus where they function, RNAP complexes are assembled at least partially in the cytoplasm. Yeast RNAPI and RNAPIII share heterodimer Rpc40-Rpc19, a functional equivalent to the αα homodimer which initiates assembly of prokaryotic RNAP. In the process of yeast RNAPI and RNAPIII biogenesis, Rpc40 and Rpc19 form the assembly platform together with two small, bona fide eukaryotic subunits, Rpb10 and Rpb12. We propose that this assembly platform is co-translationally seeded while the Rpb10 subunit is synthesized by cytoplasmic ribosome machinery. The translation of Rpb10 is stimulated by Rbs1 protein, which binds to the 3′-untranslated region of *RPB10* mRNA and hypothetically brings together Rpc19 and Rpc40 subunits to form the αα-like heterodimer. We suggest that such a co-translational mechanism is involved in the assembly of RNAPI and RNAPIII complexes.

## Introduction

**FIGURE 1:**
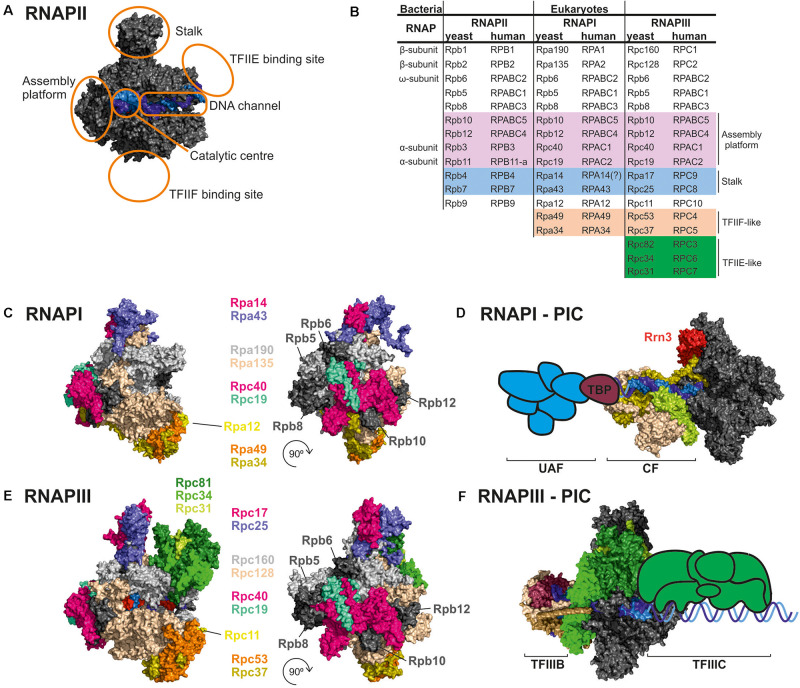
Comparison of RNAPI and RNAPIII structures and transcription factors. **(A)** General architecture of RNAPII, consisting of the catalytic core and stalk. RNAPII core consists of a DNA binding channel, catalytic center, and assembly platform. RNAPII binds multiple transcription factors (TFs). Some TFs are homologous to additional subunits of specialized RNAPs (i.e., TFIIF). **(B)** Subunit composition of eukaryotic RNAPs. Human nomenclature is shown for comparision. Please note that C-terminal region of Rpa49 subunit harbors a “tandem winged helix” which is predicted in TFIIE and that human RNAPIII RPC7 subunit is coded by two isoforms α and β. The question mark indicates name unconfirmed. **(C)** Subunit composition of yeast RNAPI. **(D)** Model of the RNAPI pre-initiation complex, showing an early intermediate with visible Rrn3 and core factor (CF). TATA-binding protein (TBP) and upstream-associated factor (UAF) are added schematically. **(E)** Subunit composition of yeast RNAPIII. **(F)** Atomic model of RNAPIII pre-initiation complex with TFIIIB. The Rpc82/34/31 heterotrimer is involved in initiation and marked in green as in E. TFIIIC is added schematically. PDB: 5C4X, 5FJ8, 4C3J, 6EU0, and 6TPS (Fernández-Tornero et al., 2013; Barnes et al., 2015; Hoffmann et al., 2015; Abascal-Palacios et al., 2018; Pilsl and Engel, 2020).

Gene expression is one of the most fundamental processes in all domains of life. DNA is transcribed to RNA by complex machinery, the core component of which is RNA polymerase (RNAP). Both bacteria and archaea have single RNAPs, multiprotein complexes that originated from two-barrel RNA polymerase enzymes and present a high degree of similarity, including other core subunits and various auxiliary factors (Figure 1; Werner and Grohmann, 2011; Fouqueau et al., 2017). Eukaryotes have at least three RNAPs that transcribe nuclear genes. RNAPII, which transcribes messenger RNAs (mRNAs), is most similar to archaeal RNAP (Werner and Weinzierl, 2002). RNAPI and RNAPIII specialize in transcribing highly abundant non-coding RNAs, including ribosomal RNA (rRNA) and transfer RNA (tRNA).

The mechanisms that allowed for the evolution of RNAPI and RNAPIII remain unknown. Recent findings suggest that eukaryotic cells evolved from Asgard archaea, which are able to form a stable interface with bacteria (Zaremba-Niedzwiedzka et al., 2017; Imachi et al., 2020). This evolutionary step may be associated with the establishment of the compact nucleoprotein organization which formed pre-nucleus and thus reflect a physical limitation that is available for transcription.

## Overview of Transcription Systems

Yeast RNAPII transcribes various different transcripts, mainly mRNAs, the abundance of which spans slightly more than two orders of magnitude (Lahtvee et al., 2017). Transcripts undergo various co-transcriptional modifications, including 5′ capping, splicing, cleavage, and polyadenylation. RNAPI transcribes only one 7-kb-long pre-rRNA, a polycistronic transcript from ∼150 rDNA repeats in yeast. RNAPI undergoes general regulation, and its transcriptional output is regulated by the availability of rDNA repeats (Wittner et al., 2011; Turowski, 2013). RNAPIII transcribes short, abundant non-coding RNA, including tRNA and 5S rRNA (Leśniewska and Boguta, 2017).

Transcription initiation by RNAPII depends on multiple transcription factors (TFs), including TATA-binding protein (TBP), TFIIA, TFIIB, TFIID, TFIIE, TFIIF, and TFIIH (Schier and Taatjes, 2020). RNAPI and RNAPIII initiate transcription *in vivo* by utilizing dedicated TFs. RNAPI utilizes Rrn3, TBP, core factor (CF), and upstream-associated factor (UAF) (Figures 1C,D; Albert et al., 2012). The RNAPIII preinitiation complex includes binding of internal promoters by multisubunit TFIIIC followed by recruitment of TFIIIB (consisted of TBP, Brf1/Brf2, and Bdp1) to the transcription start site (Figures 1E,F). TBP is involved in transcription initiation by all three RNAPs, and is recruited to TATA-containing as well to TATA-less promoters, while Brf1 is functionally related to the TFIIB (Turowski and Tollervey, 2016; Ciesla et al., 2018; Ramsay and Vannini, 2018). TFs play a key role in transcription initiation which requires opening of the DNA double helix and directing initial RNA synthesis. When formed, the DNA-RNA-RNAP ternary complex has extraordinary stability (Cai and Luse, 1987; Churchman and Weissman, 2011). Biochemical data clearly indicate that all RNAPs have high affinity for an RNA-DNA hybrid (Greive and von Hippel, 2005), confirming that opening of the DNA double helix is a key step in transcription initiation for all eukaryotic RNAPs whereas additional RNAP-specific factors account for differences in promoter recognition and gene-class specific regulation.

Research during the last decade revealed new mechanisms that are important for the regulation of eukaryotic transcription. RNAPII was shown to transcribe nearly the entire genome at a low level, a process referred to as pervasive transcription (Churchman and Weissman, 2011; Milligan et al., 2016). Many RNAPII promoters are bidirectional, and antisense transcription is common. This is in marked contrast to RNAPI and RNAPIII transcription, which uses very specific promoters and remains unidirectional (Turowski et al., 2016, 2020; Clarke et al., 2018). Finally, transcription is regulated by the local concentration of TFs and three-dimensional chromatin organization (Hnisz et al., 2017). A high number of very weak, multivalent interactions within transcription preinitiation complexes may lead to liquid-liquid phase separation. This phenomenon was previously reported for yeast RNAPI and pre-rRNA transcription and processing (Lafontaine, 2019). Recently, phase separation was demonstrated to drive chromatin function in the human genome (Cook and Marenduzzo, 2018; Sabari et al., 2018; Frottin et al., 2019; Brackey et al., 2020).

## RNA Polymerase Structure: Similarities and Differences

There is remarkable structural and functional conservation among RNAP enzymes in all eukaryotes, from yeast to man. RNAPI and RNAPIII are homologous to RNAPII, but their structures incorporated additional subunit homologs to RNAPII TFs (Figures 1A,B). The majority of subunits are encoded by independent, RNAP-specific genes. Two subunits, Rpc40 and Rpc19, are homologous to bacterial α and shared between RNAPI and RNAPIII (Wild and Cramer, 2012). Moreover, all three eukaryotic RNAPs share five relatively small subunits: Rpb5, Rpb6, Rpb8, Rpb10, and Rpb12. Four subunits common for RNAPI and RNAPIII, Rpc40, Rpc19, Rpb10, and Rpb12, form a subcomplex called the assembly platform corresponding to the assembly platform that was defined for archaeal RNAP (Werner et al., 2000; Werner and Weinzierl, 2002). All RNAPs contain the two largest subunits that are homologous to bacterial β and β′ and slightly vary in size. For RNAPI, these are Rpa190 and Rpa135. For RNAPIII, these are Rpc160 and Rpc128. Both RNAPI and RNAPIII lack the long unstructured C-terminal domain (CTD) that is present in Rpb1, the largest subunit of RNAPII. The CTD is responsible for binding and orchestrating many RNA processing factors, such as capping enzymes or the spliceosome, and its role is tightly coupled to phosphorylation status of the CTD (Hsin and Manley, 2012). Moreover, the CTD was shown to regulate RNAPII clustering *via* a phase separation mechanism (Boehning et al., 2018).

The RNAPII Rpb4/7 (stalk) subcomplex interacts with Rpb1 directly and *via* an Rpb6 interaction (Armache et al., 2005). Interestingly, Rpb6, a subunit that is common to all three RNAPs and homologous to a small ω subunit of bacterial RNAP, participates in anchoring stalk homologs in RNAPI and RNAPIII (i.e., the heterodimers Rpa14/43 and Rpc17/25, respectively (Minakhin et al., 2001; Jasiak et al., 2006; Engel et al., 2013; Fernández-Tornero et al., 2013).

In contrast to RNAPII, specialized RNAPs incorporated TFIIF-like heterodimers as stable Rpa49/34 subunits for RNAPI and Rpc37/53 subunits for RNAPIII. Additionally, the C-terminal region of Rpa49 forms a “tandem winged helix” domain that is predicted in TFIIE (Geiger et al., 2010). The Rpa49/34 heterodimer plays a role in transcription initiation and interactions with the TF Rrn3 (Beckouet et al., 2008; Albert et al., 2011). Furthermore, RNAPIII contains a heterotrimeric subcomplex, Rpc82/34/31, that is similar to TFIIE and crucial for transcription initiation (Fernández-Tornero et al., 2007).

Another interesting feature of specialized RNAPs is incorporation of the TFIIS zinc-finger domain into polymerase subunits (Ruan et al., 2011; Khatter et al., 2017). This domain is responsible for the endonucleolytic cleavage of the nascent RNA 3′ end. In RNAPI and RNAPIII this domain fuses with Rpa12 and Rpc11 subunits, respectively. Therefore, specialized RNAPs are predicted to more effectively release from polymerase backtracking. In summary, the permanent recruitment of TFs might contribute to the efficiency of RNAPI and RNAPIII that is fundamental for optimization of the cell growth rate.

Finally, RNAPI incorporated unique features that allow complex dimerization. The dimerization of RNAPI has been shown for *S. cerevisiae* and *S. pombe*, suggesting that this is a conserved phenomenon. A homodimer of RNAPI is assembled in response to environmental stress, such as nutrient deprivation. This mechanism is reversible and can also be induced by perturbations in the ribosome biogenesis pathway, suggesting that homodimer assembly may be a storage mechanism of RNAPI (Torreira et al., 2017; Heiss et al., 2021).

The specialization of RNAP machinery appears to be a driver upon the archaea-to-eukaryote transition. Nevertheless, the incorporation of TFs may suggest an additional mechanism. We speculate that limited space within a crowded environment of the pre-nucleus transformed transient interactions into the stable incorporation of TFs into structures of RNAPI and RNAPIII. In fact, archaeal general TFB binds upstream protein coding genes but is depleted upstream the rRNA, indicating that differences between the occupancy of TFs between rRNA and mRNA transcription units are also present in archaea (Smollett et al., 2017). Additionally, ribosomal components loop together in archaeal chromatin, suggesting the spatial organization of ribosome biogenesis (Takemata and Bell, 2021). Therefore, we suggest that spatial organization of the eukaryotic genome promoted the evolution of RNAP-specific and co-evolution of specific TFs. Ultimately, the evolution of specialized transcription machinery allowed the optimal use of limited space in the nucleus organized by chromatin.

## Assembly of RNAPI and RNAPIII

Detailed knowledge of the structures of yeast RNA polymerases contrasts with the incomplete information on the control of their assembly. A hypothetical model of RNAPI and RNAPIII assembly is based on the relatively well-recognized assembly pathway of bacterial RNAP (Ghosh et al., 2001; Kannan et al., 2001; Patel et al., 2020). The initial complex is formed by two α-like subunits, Rpc40 and Rpc19 (Wild and Cramer, 2012). As supported by genetic data, formation of the Rpc19/40 heterodimer additionally involves a small common subunit, Rpb10, which has no equivalent in the prokaryotic enzyme. Mutations of the conserved motif of Rpb10 lead to a complete depletion of the largest RNAPI subunit (Rpa190) suggesting that the mutant enzyme is not properly assembled (Gadal et al., 1999).

Rbp10 overexpression suppresses conditional *rpc40* and *rpc19* mutations that prevent RNAPIII assembly (Lalo et al., 1993) as well as a conditional *rpc128*-*1007* mutant that is located in the Rpc128 subunit near contact points for the association between Rpc128 and Rpc40 contact points (Cieśla et al., 2015). Rpb10 may function in the RNAP assembly platform by acting as structural adaptor between the α-like dimer Rpc40-Rpc19 and catalytic β-like subunit Rpc128. Such a role was suggested for the archaeal subunit N, which is homologous to yeast Rpb10 (Werner et al., 2000). Essential function in the formation of assembly platform of all RNAPs, by bridging between the Rpc40-Rpc19-Rpb10 subcomplex (or Rpb3-Rpb11-Rpb10 in RNAP II) and the β-like subunit, was postulated for Rpb12 (Cramer et al., 2000). A role of Rpb12 in RNAPIII assembly was also supported by earlier genetic data (Rubbi et al., 1999).

The existence of intermediate complexes in the process of yeast RNAP assembly was suggested by the mass spectrometry analysis of RNAPIII disassembly (Lorenzen et al., 2007; Lane et al., 2011). These analyses revealed two stable subcomplexes, Rpc128-Rpc40-Rpc19-Rpb12 and Rpc160-Rpb8-Rpb5. In addition to Rpb10, other small subunits also contribute to the association of these macromolecular assembles (Minakhin et al., 2001; Mirón-García et al., 2013). Although common to all RNAPs, the small subunits may have distinct functions in the assembly of each RNAP, thereby providing an interaction platform for other molecules (Voutsina et al., 1999).

According to an existing model (Wild and Cramer, 2012), eukaryotic RNAP enzymes are at least partially assembled in the cytoplasm and then imported to the nucleus as a complex with specific adaptor proteins. A set of RNAPIII subunits exhibit coordinated nuclear import, indicating that the RNAPIII core is assembled in the cytoplasm, with additional components that bind in the nucleus (Hardeland and Hurt, 2006). This suggests that the specific subcomplexes, particularly Rpc82-Rpc34-Rpc31, would only bind the core in the nucleus (Hardeland and Hurt, 2006). Interestingly, efficient RNAPIII assembly requires sumoylation of the Rpc82 subunit, which is RNAPIII-specific (Chymkowitch et al., 2017).

Several auxiliary factors, originally implicated in RNAPII assembly and nuclear import and subsequently shown to be common to RNAPI and RNAPIII were described in another article published in the same issue by Navarro and colleagues. Here we focus on the Rbs1 protein, a candidate RNAPIII assembly/import factor, which was identified in a genetic screen for suppressors of the RNAPIII assembly mutant *rpc128*-*1007* (Cieśla et al., 2015). Genetic suppression correlated with an increase in the stability of RNAPIII subunits and an increase in their interaction. Additionally, Rbs1 physically interacts with a subset of RNAPIII subunits (i.e., Rpc19, Rpc40, and Rpb5) and the exportin Crm1. We postulated that Rbs1 binds to the RNAPIII complex or subcomplex and facilitates its translocation to the nucleus. Following dissociation from RNAPIII in the nucleus, Rbs1 is exported back to the cytoplasm in complex with Crm1 (Cieśla et al., 2015).

It is reasonable that the Rbs1 function in RNAP assembly is not limited to RNAPIII. Rbs1 interacts with Rpc19 and Rpc40 subunits common to RNAP I and RNAPIII and Rpb5, a component of all three RNAPs (Cieśla et al., 2015). Moreover, Rpb5 participates in the assembly of all three polymerases mediated by Bud27 (Mirón-García et al., 2013).

Genetic and functional suppression of the RNAPIII assembly defect by Rbs1 correlated with higher levels of *RPB10* mRNA and Rpb10 protein. This regulatory mechanism, however, relies on the control of steady-state levels of *RPB10* mRNA by Rbs1 protein, which interacts with the 3′-untranslated region (UTR) of this transcript (Cieśla et al., 2020).

By exploring specific features of the Rbs1 protein sequence, we identified two regions: a highly ordered N-terminal region that comprises two RNA-interacting domains (R3H and SUZ) and a mostly disordered C-terminal region with a prionogenic (aggregation-promoting) sequence. Investigations of possible roles of these regions in *RBS1* led to the conclusion that the R3H domain was essential for suppressing both genetic and molecular phenotypes of the *rpc128*-*1007* mutation and function of Rbs1 protein in RNAPIII assembly, whereas the role of the prionogenic domain remains unknown (Cieśla et al., 2020).

By applying ultraviolet crosslinking, we identified the transcriptome-wide binding of Rbs1, which predominately targets 3′-UTRs of mRNAs. The list of high-confidence Rbs1 targets included *RPB10* mRNA and *RPC19* mRNA, which encodes Rpc19, another subunit involved in formation of the assembly platform for RNAPIII (Cieśla et al., 2020).

Notably, homologs of Rbs1 have been identified in other eukaryotes, including the human proteins R3H domain protein 2 (R3HDM2) and cyclic adenosine monophosphate-regulated phosphoprotein 21 (ARPP21), which are also known to interact with mRNA (Castello et al., 2012; Rehfeld et al., 2018).

## Model of Co-Translational Formation of the Rpc40-Rpc19-Rpb12-Rpb10 Assembly Platform

**FIGURE 2:**
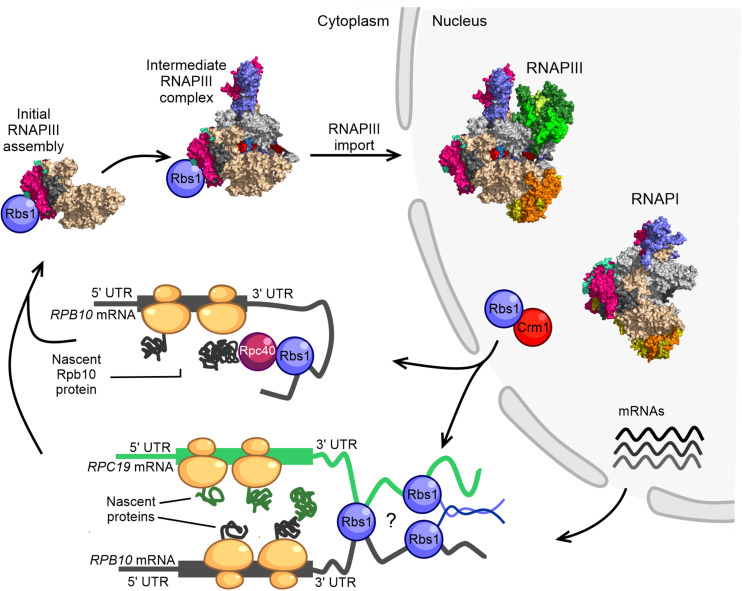
Model of RNAPIII biogenesis in the yeast *Saccharomyces cerevisiae*. The control of Rpb10 expression and role of Rpb10 in assembly of the RNAP III complex are connected *via* a regulatory loop that involves Rbs1 protein. Possible ways in which subunits of the RNAPIII intermediate complex are brought together for co-translational assembly are shown. The initial step of RNAPIII assembly in *Saccharomyces cerevisiae* occurs in the cytoplasm. Formation of the intermediate Rpc128-Rpc40-Rpc19-Rpb12-Rpb10 subcomplex is seeded co-translationally while the Rpb10 subunit is being synthesized by cytoplasmic ribosomes. Rbs1 is an RNA binding protein that stimulates the translation of Rpb10 protein through an interaction of the R3H domain with the 3′-UTR in *RPB10* mRNA. Rpb10 brings together the Rpc19 and Rpc40 subunits to form the α-like heterodimer. One possibility is that Rbs1 binds and recruits the mature Rpc40 subunit to the 3′-UTR of *RPB10* mRNA, which undergoes translation. The Rpc40-Rbs1 interaction has been previously demonstrated by co-immunoprecipitation. Alternatively, Rbs1 protein directly bridges *RPB10* mRNA and *RPC19* mRNA. A fully folded subunit that formed on one mRNA was recently shown to detach from its ribosome and interact with a nascent protein on another mRNA (Cieśla et al., 2015, 2020).

Based on established interactions between Rbs1 and the subunits of RNAPIII (Cieśla et al., 2015, 2020), we propose a co-translational mechanism of formation of the early-stage assembly intermediate of the RNAPIII complex and potentially also RNAPI (Figure 2). According to our hypothesis, RNAPIII assembly might be seeded while the Rpb10 subunit of the enzyme core is being synthesized by cytoplasmic ribosome machinery. This assembly pathway would be proceeded by the co-translational association of other subunits, including Rpc19 and Rpc40 (Figure 2), to build an initial assembly subcomplex that is common to RNAPI and RNAPIII. Currently unknown, however, is how the Rpc40-Rpc19-Rpb10 complex discriminates among Rpc128 and Rpa135 proteins to proceed with formation of the RNAPIII and RNAPI assembly platform.

Co-translational assembly has been reported for several multisubunit complexes (e.g., TFIID, TREX-2, SAGA, and fatty acid synthase; Kamenova et al., 2019; Schwarz and Beck, 2019; Shiber et al., 2018) but has not yet been considered for RNA polymerases. Our hypothesis is in line with the idea that that co-translational subunit association is likely to be a general principle in yeast and mammalian cells as an efficient assembly pathway in eukaryotes (Shiber et al., 2018).

For RNAPIII, we propose two plausible models that are not necessarily mutually exclusive and could be applicable to RNAPI (Figure 2). In the first model, the long 3′-UTR of *RPB10* acts as a scaffold to recruit Rbs1 that is associated with another RNAPIII subunit (e.g., Rpc40) that interacts with Rbs1 through co-immunoprecipitation (Cieśla et al., 2015) to the site of Rpb10 translation. This facilitates the association of this subunit with the newly translated Rpb10 to form the RNAP assembly platform subcomplex. Such a scenario corresponds to a sequential assembly model, in which RNA-binding protein recruits a fully folded subunit to the 3′-UTR of mRNA that encodes the second subunit that undergoes translation. The 3′-UTR regions can act as scaffolds for RNA binding proteins that serve as adaptors to deliver preferred proteins to the site of translation (Berkovits and Mayr, 2015). The sequential assembly pathway has been proposed for the co-translational assembly of TAF8-TAF10 subunits of TFIID and TAF1-TBP assembly. TAF10 binds the nascent TAF8 subunit, and TAF10 protein co-localizes with *TAF8* mRNA in cytoplasmic foci (Kamenova et al., 2019).

In the second model, *RPB10* and *RPC19* mRNAs are bridged together by Rbs1, which interacts with 3′-regulatory regions of both transcripts (Cieśla et al., 2020). Additionally, unstructured parts of Rbs1 may facilitate interaction among Rbs1 molecules allowing the Rpb10 and Rpc19 subunits to be translated in proximity to each other, thereby enabling their co-translational interaction (Figure 2). A simultaneous model has been proposed for the co-translational assembly of TAF6 and TAF9 subunits of the transcription factor TFIID (Kamenova et al., 2019). Physical linkage of the two mRNAs could also be accomplished by their co-localization in phase-separated compartments that allow translation at defined subcellular locations (Mayr, 2018).

Rbs1 exhibits all characteristics of the postulated protein that bridges mRNA. The two RNA-interacting domains, R3H and SUZ, have been identified in the sequence of Rbs1 protein, and this sequence also contains a prionogenic, disordered region. The specific mRNA motifs and potential effect of Rbs1 binding on the translation of these targets need to be determined. R3H likely cooperates with the SUZ domain in the recognition of specific mRNA targets and bridging them into proximity with each other. A disordered region of Rbs1 may be involved in multivalent interactions that bring Rbs1-associated mRNAs together. Such an Rbs1-mediated co-localization of mRNAs would allow them to be translated at defined subcellular locations.

## Stoichiometry of Subunits of Specialized RNAPs

The assembly platform Rpc40-Rpc19-Rpb10-Rpb12 is shared between yeast RNAPI and III what arises question about the stoichiometry of RNAPs subunits during the assembly pathway. The absolute quantification of yeast proteins indicated that RNAPI and RNAPII are present in 5,000 copies per cell, whereas RNAPIII is present in 2,500 copies (Turowski et al., 2020). Consequently, common subunits are shared between RNAPI, RNAPII, and RNAPIII in a 2:2:1 ratio. RNAPI and RNAPIII share an assembly platform that contains the Rpc19 and Rpc40 subunits and two additional subunits (Rpb10 and Rpb12) among the five common subunits. Both specialized RNAPs utilize the assembly platform, sharing RNAPI:RNAPIII in a 2:1 ratio. The platform is attached *via* the second largest subunit Rpa135 to RNAPI and *via* Rpc128 to RNAPIII. Limited data suggest a difference in binding strength at this stage. A biochemical disassembly approach demonstrated that RNAPI disassembles the platform from the dimer of the two largest subunits, Rpa135 and Rpa190, whereas RNAPIII disassembles the interface between the two largest subunits before detachment of the assembly platform (Lane et al., 2011). This suggests that the Rpc128-platform interaction might be stronger than the interaction between the two largest subunits. This would be in contrast to RNAPI, in which the interaction with the two largest subunits would be stronger than the interaction with the platform. In the consequence, a common assembly platform could be preferentially incorporated by less abundant RNAPIII.

## Discussion

Despite recent progress, the RNAP assembly process remains poorly described. Knowledge about its basic mechanism is necessary to ask more detailed questions about disease and developmental biology. The structure of human RNAPI awaits to be determined. Recently published structures of human RNAPIII revealed a high level of conservation (Ramsay et al., 2020; Li et al., 2021; Wang et al., 2021). Moreover, mutations of specialized RNAPs lead to genetic disorders, such as Treacher-Collins syndrome and hypomyelinating leukodystrophy (Ramsay et al., 2020; Girbig et al., 2021), demonstrating the requirement for precise coordination among all three RNAPs and their assembly. Research on RNAPIII assembly in yeast focused on *rpc128-1007* mutations that disturbed the interface between the Rpc128 and Rpc40 subunits. Interestingly, multiple disease-associated mutations of human RNAPIII subunits tend to cluster within the region of the RNAPIII assembly platform, suggesting that defects in RNAPIII biogenesis may have severe health consequences (Ramsay et al., 2020; Girbig et al., 2021). Further studies of RNAP assembly should reveal additional factors that are involved in this process and improve our understanding of this vital pathway.

## Author Contributions

Both authors listed have made a substantial, direct and intellectual contribution to the work, and approved it for publication.

## Conflict of Interest

The authors declare that the research was conducted in the absence of any commercial or financial relationships that could be construed as a potential conflict of interest.
